# Inflammatory and coagulatory markers and exposure to different size fractions of particle mass, number and surface area air concentrations in Swedish iron foundries, in particular respirable quartz

**DOI:** 10.1007/s00420-019-01446-z

**Published:** 2019-06-04

**Authors:** Håkan Westberg, Alexander Hedbrant, Alexander Persson, Ing-Liss Bryngelsson, Anders Johansson, Annette Ericsson, Bengt Sjögren, Leo Stockfelt, Eva Särndahl, Lena Andersson

**Affiliations:** 1 Department of Occupational and Environmental Medicine, Faculty of Medicine and Health, Örebro University, 70182 Örebro, Sweden; 2 Department of Medical Sciences, School of Medicine and Health, Örebro University, 701 82 Örebro, Sweden; 3 Inflammatory Response and Infection Susceptibility Centre (iRiSC), Örebro University, 701 82 Örebro, Sweden; 4 Work Environment Toxicology, Institute of Environmental Medicine, Karolinska Institutet, 171 77 Stockholm, Sweden; 5 Department of Occupational and Environmental Medicine, University of Gothenburg, PB 414, 405 30 Göteborg, Sweden; 6 Department of Occupational and Environmental Medicine, Örebro University Hospital, 701 85 Örebro, Sweden

**Keywords:** Inflammatory markers, Particle mass, Particle number, Particle surface area, Respirable quartz, Iron foundries

## Abstract

**Purpose:**

To study the relationship between inhalation of airborne particles and quartz in Swedish iron foundries and markers of inflammation and coagulation in blood.

**Methods:**

Personal sampling of respirable dust and quartz was performed for 85 subjects in three Swedish iron foundries. Stationary measurements were used to study the concentrations of respirable dust and quartz, inhalable and total dust, PM10 and PM2.5, as well as the particle surface area and the particle number concentrations. Markers of inflammation, namely interleukins (IL-1β, IL-6, IL-8, IL-10 and IL-12), C-reactive protein, and serum amyloid A (SAA) were measured in plasma or serum, together with markers of coagulation including fibrinogen, factor VIII (FVIII), von Willebrand factor and d-dimer. Complete sampling was performed on the second or third day of a working week after a work-free weekend, and follow-up samples were collected 2 days later. A mixed model analysis was performed including sex, age, smoking, infections, blood group, sampling day and BMI as covariates.

**Results:**

The average 8-h time-weighted average air concentrations of respirable dust and quartz were 0.85 mg/m^3^ and 0.052 mg/m^3^, respectively. Participants in high-exposure groups with respect to some of the measured particle types exhibited significantly elevated levels of SAA, fibrinogen and FVIII.

**Conclusions:**

These observed relationships between particle exposure and inflammatory markers may indicate an increased risk of cardiovascular disease among foundry workers with high particulate exposure.

## Introduction

In 2016, Swedish foundries produced 292,000 tons, of which 209,000 tons were from the iron foundries that collectively employ 7500 foundry workers (Nayström 2018). In the foundry industry, concerns about aerosol exposure have primarily related to respirable quartz (CAS no.14080-60-7), mostly due the risk of silicosis (Rees and Weiner 1994; Rosenman et al. 1996; Schudel 1960) and lung cancer (Sherson et al. 1991; Andjelkovich et al. 1990; Rodriguez et al. 2000; Westberg and Bellander 2003; Xu et al. 1996). The working environment of iron foundries has also been linked to elevated risk of cardiovascular diseases (Koskela et al. 2005; Palda 2003; Fan et al. 2018) and mortality (Vena et al. 1985; Decoufle and Wood 1979; Silverstein et al. 1986; Yoon and Hahn 2014).

Exposure to ambient airborne particulate matter inhalation has been associated with increases in mortality and hospital admissions due to cardiovascular diseases, and mechanistic evidence from animal and human studies suggests that these outcomes are associated with systemic inflammatory responses. Other potentially relevant processes include disturbances in the autonomic nervous system, and the direct effects of particles reaching the systemic circulation are other potential pathways (Brook et al. 2010).

The inflammatory causal link between inhalation of particulate matter and cardiovascular disease was first proposed in the mid-1990s (Seaton et al.^1995^; Sjögren 1997). Later publications revealed some of the mechanisms underpinning this link, notably the formation of the NLRP3 inflammasome upon exposure of intracellular receptors to particles, which leads to the activation of the enzyme caspase-1, and subsequently of interleukin IL-1β and other cytokines (Dagenais et al. 2012; Dostert et al.^2008^; Peeters et al. 2013).

Several inflammatory markers, including IL-6, C-reactive protein (CRP), and fibrinogen, are established risk factors for cardiovascular disease (Danesh et al. 2000, 2005, 2008). Serum amyloid A (SAA) has also been proposed as a risk factor for cardiovascular disease as a component of a battery of several biomarkers (Arant et al. 2009). Quartz exposure has been linked to increased levels of TNF-α, IL- 6 and IL-1β, according to a review of animal studies (Kawasaki 2015). In a series of studies, we have determined exposure responses for various inflammatory markers with respect to particle exposure in mechanical and welding workshops, cement plants, aluminium foundries and in the pulp and paper industry (Ohlson et al. 2010; Westberg et al. 2016). Here, we extend this work to cover iron foundries.

The purpose of this study was to investigate a possible exposure–response relationship between particulate exposure (quantified in terms of levels of respirable dust and quartz, and particle mass, number and surface area concentrations in air) and markers of inflammation and coagulation.

## Methods

### Study group

The study was performed at three Swedish iron foundries—a large foundry employing 440 workers and two smaller ones employing 90 and 25 workers, respectively. The yearly outputs of these foundries amount to 100,000, 25,000 and 2000 tons of castings, respectively. These castings are mainly based on iron and grey iron alloys, and include products such as motor heads and blocks for trucks, large components for the wind power industry and medium- and small-sized lego castings. The jobs and departments included in the study were sand preparation, core making, moulding, casting, shake-out operations, fettling, inspection, maintenance and repair. The sand used for moulding was mostly green sand (75% SiO_2_, 6% carbon black, 5–6% bentonite and 13–14% water), but chemical binders such as furan (furfuryl alcohol, urea, phenol, formaldehyde, *p*-toluenesulphonic acid or phosphoric acid) and silicate ester (sodium meta silicate and organic ester) were added to the silica sand. The tasks performed at the foundries are mechanized and manual moulding and casting. The most commonly used core binder was epoxy–SO_2_ and sodium silicate, but coldbox (isocyanate-MDI, polyol, phenol formaldehyde) resin with an amine as catalytic agent was also used for this purpose (American Foundry Society 2018). Eighty-five foundry workers (80 men and 5 women), most of whom worked day shifts, participated in the study. Their ages ranged from 21 to 67 years, with a mean of 43, and the participants were evenly distributed over 10-year age groups. They had worked in the same workplace for 12 years on average, with a maximum of 45 years’ continuous employment. Eighty-three participants reported their smoking habits: 17 were current smokers, 18 ex-smokers and 48 were never smokers.

### Study design

Six separate 4-day measurement campaigns were conducted between March 2015 and September 2016. During each campaign, aerosol and blood sampling was performed on the second or third working day after a work-free weekend. Blood sampling was performed twice for each subject, at work: once in the afternoon (3–4.30 p.m.) on the first day of the campaign, and again 2 days later (i.e., on day 4 or 5). The post-shift sampling design was intended to control for circadian variation. All participants completed a questionnaire containing items relating to their current and previous working conditions, height and weight, smoking habits, medication, symptoms of respiratory irritation and infections or other inflammatory conditions that could affect the levels of the studied biomarkers. All participants were at work during our investigation. Seven participants reported chronic diseases such as thyroid imbalance, type 2 diabetes, scoliosis, hypertension, depression, asthma or COPD.

### Aerosol measurements and quartz analysis

The respiratory tract comprises three regions: the extrathoracic region (including the nose and throat), the thoracic region and the alveolar region. Particles that enter the extrathoracic region constitute the inhalable fraction, those that penetrate into the thoracic region constitute the thoracic fraction and particles deposited into the alveolar region constitute the respirable fraction. The PM_10_ and PM_2.5_ fractions, which are discussed widely in environmental analyses, correspond to the thoracic and respirable fractions, respectively (Vincent 2005).

The participants’ respirable and quartz dust exposure was determined by personal sampling on the 2nd or 3rd day after a work-free weekend, on the same day as the first blood sample collection. Personal sampling of respirable dust was performed by collecting 8-h full shift samples according to standard protocols using GSP samplers operating at 3.5 l/min (HSE 2000). A minority of samples were collected using SKC AircheckXR5000 or GSA SG5100 sampling pumps. Respirable crystalline quartz and cristobalite were determined by X-ray diffraction (NIOSH 1994a) (modified).

Stationary measurements (area measurements) were acquired using measuring rigs. These measurements were conducted in the departments where the participants worked during their shifts and included sampling of respirable dust and quartz using the same techniques as for the personal samples. Total dust was determined by collecting 8-h full shift samples according to a modified gravimetric method (NIOSH 1994b) using open-faced 25 mm filter cassettes (OFC) with an air flow of 2.0 l/min. Respirable dust was determined using SKC HD aluminium cyclones with 25 mm filters, operating at an air flow of 2.5 l/min (HSE 2000). Air concentrations of PM_10_ and PM_2.5_ were determined using a ChemPass system operating at 1.8 l/min (EN 1999, 2005). For PM_1_, a Dusttrak DRX Monitor 8533 was used. Particle surface area air concentrations were determined using an A-trak 9000 (Aero Trak Nanoparticle Aerosol Monitor) instrument, which determines the particle surface area for particles in the size range 10–1000 nm in real time. Particle number concentrations were measured based on particles in the 20–1000 nm size range, using a real-time aerosol monitoring instrument (P-Trak 8525 Ultrafine Particle Counter).

### Markers of inflammation and coagulation

The blood samples were centrifuged and stored at − 70° in a biobank (Dnr 13OLL718-5). The concentrations of several interleukins (IL-1β, IL-6, IL-8, IL-10 and IL-12) and tumor necrosis factor (TNF) were determined at the research laboratory of iRiSC, Örebro University. These measurements were performed with a Luminex 200TM recorder (12212 Technology Blvd, Austin, Texas, USA) and analyzed using the Luminex xPONENT software package. High-sensitivity CRP, fibrinogen, d-dimer and blood groups (ABO) were determined at the Clinical Research Center, Örebro University Hospital. Serum amyloid A (SAA), factor VIII (FVIII) and the von Willebrand factor (vWF) were analysed at the Department of Occupational and Environmental Medicine, Sahlgrenska University Hospital, Göteborg.

### Exposure measures

Exposure was assessed by performing personal sampling to measure concentrations of respirable dust and quartz, and by performing stationary measurements in each department that served as proxies for the total and inhalable dust, PM_10,_ PM_2.5_, PM_1_, particle surface area and particle number concentrations in the air. The 28 participants working in known high-exposure jobs (particularly shake-out and fettling operations) wore respirators while working. To account for the respirators’ effects, the raw measurements for these subjects were adjusted to better assess their true exposure. Specifically, it was assumed that these workers experienced zero exposure while wearing a respirator mask; their exposure during the remainder of their shifts was estimated based on either personal sampling or the measured background concentrations for the departments they worked in while not wearing respirators.

Three exposure measures were computed: the non-adjusted exposure (*C*_average_) as determined by personal sampling; the average adjusted (*C*_average adjusted_) exposure based on the assumption of null exposure when using a respirator and the uncorrected exposure determined by personal sampling for the rest of the day; and the background-adjusted (*C*_background adjusted_) exposure based on the assumption of null exposure when using a respirator and the background exposure determined by stationary sampling in the relevant departments.

1. Non-adjusted exposure = *C*_average_
2. Average adjusted exposure *C*_average adj_ = (*c*_(1) _× *t*_(1)_ + *c*_(2) _× *t*_(2)_)/(*t*_(1)_ + *t*_(2)_)
3. Background-adjusted exposure *C*_background adj_ = (*c*_(1) _× *t*_(1)_ + *c*_(3) _× *t*_(2)_)/(*t*_(1)_ + *t*_(2)_)

*c*_(1)_ = exposure using respirator, assuming *c*_(1)_ = 0

*t*_(1)_ = time using respirator

*c*_(2)_ = uncorrected air concentration

*t*_(2)_ = time spent without wearing respirator

*c*_(3)_ = background air concentration for relevant departments

### Statistical analysis

For descriptive purposes, the measured aerosol concentrations, i.e., the concentrations of inhalable, total and respirable dust, respirable quartz and cristobalite, PM_10_ and PM_2.5_, as well as the particle surface area (A-Trak) and particle number concentration (P-trak) are reported as 8-h TWAs for the full workday. Standard parameters such as arithmetic means (AM), standard deviations (SD), geometric means (GM), geometric standard deviations (GSD) and ranges were calculated based on the log-normal distributions of each measurement, including the stationary samples of the aerosol mass fractions and the particle number and particle surface area concentrations (Table 1). Respirable quartz exposure by job title is presented in Table 2; the corresponding descriptions, means and ranges of the measured blood concentrations by sampling day are presented in Table 3. The analyses presented below are based on background-adjusted quartz and respirable particle data.

**Table 1:** Exposure concentration levels of respirable dust, quartz and cristobalite

Aerosol concentration levels	*N*	AM	Median	SD	GM	GSD	Min	Max
Exposure measurements								
Respirable dust (mg/m^3^)	85	0.85	0.35	1.6	0.39	3.1	0.062	9.7
Respirable quartz (mg/m^3^)	85	0.052	0.018	0.11	0.016	4.4	0.0013	0.61
Respirable cristobalite (mg/m^3^)	85	0.0037	0.0034	0.0015	0.0035	1.3	0.0028	0.014
Stationary measurements								
Inhalable dust (mg/m^3^)	36	3.2	2.3	3.9	1.6	3.9	0.045	16
Total dust (mg/m^3^)	36	3.4	2.2	4.3	1.5	4.4	0.078	16
Respirable dust (mg/m^3^)	36	0.58	0.36	0.58	0.32	3.3	0.058	1.9
Respirable quartz (mg/m^3^)	36	0.026	0.011	0.37	0.031	6.8	0.0012	0.26
Respirable cristobalite (mg/m^3^)	36	0.0034	0.0031	0.00096	0.0033	1.2	0.0029	0.0072
PM_10_ (mg/m^3^)	36	1.9	1.6	1.9	1.1	3.1	0.15	8.7
PM_2.5_ (mg/m^3^)	36	1.2	0.57	1.9	0.63	3.1	0.085	11
PM_1.0_ (mg/m^3^)^a^	35	0.35	0.2	0.41	0.27	3.7	0.018	1.7
Total particle area (A-trak) (µm^2^/cm^3^)	34	420	140	540	160	4.7	9.2	1900
Particle number ((P-trak)/cm^3^)	36	78,000	43,000	91,000	32,000	4.7	1500	360,000

Stationary concentration levels of inhalable, total and respirable dust, respirable quartz and cristobalite, PM_10_, PM_2.5_, PM_1.0_, particle surface area (A-trak) and particle number (P-trak)

*N* number of measurements, *AM* arithmetic mean, *SD* standard deviation, *GM* geometric mean, *GSD* geometric standard deviation

^a^Dusttrak

**Table 2:** Exposure measurements of respirable quartz by job title

Agent	Job title	N	AM	Median	SD	GM	GSD	Min	Max
Quartz	Sand preparation	4	0.026	0.026	0.018	0.02	2.4	0.0064	0.046
	Melting	13	0.029	0.013	0.05	0.013	3.5	0.0014	0.19
	Core making	5	0.09	0.028	0.14	0.017	12	0.0013	0.33
	Moulding	14	0.025	0.023	0.021	0.016	3.1	0.0014	0.077
	Casting	4	0.0066	0.0056	0.004	0.0057	1.8	0.0029	0.012
	Shake out	5	0.12	0.08	0.11	0.08	2.8	0.023	0.29
	Fettling and blasting	18	0.12	0.03	0.21	0.024	7.6	0.0014	0.61
	Inspection	4	0.029	0.031	0.024	0.016	4.6	0.0019	0.05
	Maintenance incl. cleaning	4	0.022	0.024	0.012	0.019	2.2	0.0059	0.035
	Transport	7	0.016	0.017	0.0086	0.013	2	0.0039	0.029
	Others	7	0.0066	0.0058	0.0042	0.0053	2.1	0.0015	0.014
	Total	85	0.052	0.018	0.11	0.016	4.4	0.0013	0.61

*N* number of measurements, *AM* arithmetic mean, *SD* standard deviation, *GM* geometric mean, *GSD* geometric standard deviation

**Table 3:** Blood concentrations of biological markers (mean, median, minimum and maximum) among 85 participants on different days

Marker	Sampling day 1					Sampling day 2				
	*N*	Mean	Median	Min	Max	*N*	Mean	Median	Min	Max
Inflammation										
TNF α (pg/ml)	85	4.65	4.52	1.94	9.62	85	5.46	5.41	1.60	11.07
IL-1β (pg/ml)	85	0.20	0.15	0.02	0.60	85	0.35	0.37	0.02	0.83
IL-6 (pg/ml)	85	1.15	0.95	0.22	4.25	85	1.50	1.26	0.31	11.20
IL-8 (pg/ml)	85	3.23	2.93	0.76	10.32	85	3.48	3.22	0.85	7.66
IL-10 (pg/ml)	85	0.67	0.61	0.31	1.52	85	0.78	0.75	0.19	2.30
IL-12 (pg/ml)	85	0.74	0.07	0.07	3.47	85	1.10	0.59	0.07	12.52
P-SAA (mg/l)	85	17.56	8.69	1.83	202.79	85	15.75	6.59	2.0	235.2
CRP (mg/l)	85	1.98	1.11	0.08	13.37	85	1.82	0.89	0.04	14.45
Coagulation										
Fibrinogen (g/l)	85	3.4	3.4	1.82	5.06	85	3.4	3.37	1.73	4.88
d-dimer (µg/ml)	85	0.37	0.35	0.35	0.90	85	0.37	0.35	0.35	0.76
vWF (kIU/l)	85	1.21	1.12	0.39	2.47	85	1.14	1.04	0.35	2.34
Factor VIII (kIU/l)	85	1.17	1.18	0.45	2.80	85	1.21	1.20	0.42	2.44

A linear mixed model was developed to describe the relationship between the different exposure metrics and the inflammatory markers. Our design and the use of mixed model made it possible to consider within- and between-worker variability based on our repeated measurements of inflammatory markers. We used a mixed model to study the variation in biomarker levels for exposure categories 1–3 (Tables 4, 5). Because the distribution of inflammatory markers was skewed, the biomarker data were log-transformed. The estimates (*β*) of the fixed effects from the model allowed us to identify the factors affecting the inflammatory markers. The equation of the final mixed model is:

$$ \begin{aligned} Y = \, & { \ln }\left( X \right) \, = \, \mu \, + \, \beta_{ 1} \left[ {\text{EXPOSURE}} \right] + \, \beta_{ 2} \left[ {\text{DAY}} \right] + \, \beta_{ 3} \left[ {\text{GENDER}} \right] + \, \beta_{ 4} \left[ {\text{BMI}} \right] \\ & \quad + \beta_{ 5} \left[ {\text{SMOKING}} \right] + \, \beta_{ 6} \left[ {\text{AGE}} \right] + \beta_{ 7} \left[ {\text{INFECTION}} \right] + \, \beta_{ 8} \left[ {\text{BLOODGROUP}} \right] + \varepsilon , \\ \end{aligned} $$

where *Y* is ln(*X*); *X* and *Y* are the measured and log-transformed inflammatory marker concentrations, respectively. *µ* is the overall average inflammatory marker concentration on the log-scale and *β*_1_ is the fixed effect of the exposure measure in question (*i*… *j*) in the given exposure group, with the lowest exposure group (exposure class 1) serving as the reference category. The exposure and stationary measurement data were categorized into three classes (with a minimum of ten workers per class) to achieve high contrast.

**Table 4:** Mixed model for inflammatory markers IL-6, IL-8, SAA and CRP, by exposure class and antilog values

Exposure	IL-6			IL-8			SAA			CRP		
	*β*	95% CI		*β*	95% CI		*β*	95% CI		*β*	95% CI	
Respirable quartz (mg/m^3^ p)												
> 0.05	**1.16**	0.83	1.62	**1.12**	0.83	1.51	**1.24**	0.57	2.68	1.12	0.52	2.40
0.01–0.05	**1.04**	0.85	1.26	**1.04**	0.87	1.24	**1.21**	0.77	1.89	1.34	0.86	2.10
< 0.01	**1**	1	1	**1**	1	1	**1**	1	1	1	1	1
Respirable dust (mg/m^3^ p)												
> 1.0	**1.06**	0.74	1.16	**1.02**	0.76	1.37	0.69	0.32	1.51	1.22	0.58	2.53
0.1–1.0	**1.06**	0.91	1.22	**1.02**	0.83	1.24	1.14	0.73	1.80	1.32	0.82	2.12
< 0.1	**1**	1	1	**1**	1	1	1	1	1	1	1	1
Inhalable dust (mg/m^3^ s)												
> 5	**1.37**	0.89	2.12	1.13	0.77	1.67	***3.22***	***1.22***	***8.47***	2.02	0.73	5.59
0.1–5	**1.19**	0.84	1.68	0.86	0.63	1.17	**1.84**	0.91	3.72	2.06	0.91	4.65
< 0.1	**1**	1	1	1	1	1	**1**	1	1	1	1	1
Total dust (mg/m^3^ s)												
> 5	1.16	0.84	1.60	1.24	0.93	1.64	**2.00**	0.91	4.43	1.05	0.49	2.24
0.1–5	0.96	0.79	1.17	0.90	0.75	1.08	**1.12**	0.72	1.73	0.96	0.59	1.55
< 0.1	1	1	1	1	1	1	**1**	1	1	1	1	1
Respirable dust (mg/m^3^ s)												
> 1.0	1.02	0.73	1.43	0.85	0.63	1.15	1.40	0.67	2.92	**1.48**	0.68	3.23
0.1–1.0	0.97	0.74	1.28	0.93	0.72	1.19	1.48	0.84	2.59	**1.31**	0.69	2.49
< 0.1	1	1	1	1	1	1	1	1	1	**1**	1	1
Respirable quartz (mg/m^3^ s)												
> 0.05	**1.07**	0.78	1.48	1.27	0.95	1.69	**1.81**	0.83	3.92	1.01	0.48	2.11
0.01–0.05	**1.06**	0.86	1.32	0.96	0.80	1.17	**1.48**	0.93	2.34	1.62	0.99	2.65
< 0.01	**1**	1	1	1	1	1	**1**	1	1	1	1	1
PM 10 (mg/m^3^ s)												
> 2.5	1.42	0.58	1.34	0.88	0.58	1.34	**1.56**	0.57	4.27	1.82	0.61	5.39
0.1–2.5	0.82	0.56	1.20	0.82	0.56	1.20	**1.50**	0.62	3.61	2.03	0.76	5.44
< 0.1	1	1	1	1	1	1	**1**	1	1	1	1	1
PM 2.5 (mg/m^3^ s)												
> 2	**1.30**	0.89	1.91	**1.29**	0.92	1.82	***2.66***	***1.11***	***6.35***	1.61	0.66	3.93
0.1–2	**1.13**	0.86	1.50	**1.00**	0.78	1.28	**1.54**	0.88	2.69	1.67	0.87	3.20
< 0.1	**1**	1	1	**1**	1	1	**1**	1	1	1	1	1
PM 1(mg/m^3^ s)												
> 0.5	1.01	0.72	1.43	1.13	0.83	1.54	***2.57***	***1.15***	***5.73***	**1.40**	0.62	3.13
0.1–0.5	1.03	0.84	1.26	0.88	0.74	1.06	**1.21**	0.78	1.86	**1.07**	0.66	1.74
< 0.1	1	1	1	1	1	1	**1**	1	1	**1**	1	1
Particle surface area (µm^2^/cm^3^ s)												
> 1000	1.19	0.87	1.64	1.04	0.79	1.39	**1.55**	0.76	3.19	**1.35**	0.64	2.84
100–1000	1.03	0.84	1.26	0.84	0.70	1.01	**1.33**	0.85	2.08	**1.23**	0.76	2.00
< 100	1	1	1	1	1	1	**1**	1	1	**1**	1	1
Particle number (nr/10^4^ cm^3^ s)												
> 10	1.09	0.85	1.38	0.83	0.66	1.03	1.13	0.68	1.88	1.20	0.68	2.12
2–10	0.98	0.79	1.23	0.91	0.75	1.12	1.70	1.05	2.75	1.32	0.78	2.24
< 2	1	1	1	1	1	1	1	1	1	1	1	1

*β* fixed effect by exposure class, 95% CI (confidence interval)

Bold values indicate exposure response, both significant and nonsignificant, and italic values significant in the high exposure class

*p* personal sampling, *s* stationary sampling

**Table 5:** Mixed model for coagulatory markers, fibrinogen, vWF, factor VIII, by exposure class and antilog values

Exposure	Fibrinogen			vWF			Factor VIII		
	*β*	95% CI		*β*	95% CI		*β*	95% CI	
Respirable quartz (mg/m^3^ p)									
> 0.05	1.04	0.90	1.21	**1.06**	0.81	1.40	**1.06**	0.85	1.33
0.01–0.05	1.06	0.97	1.15	**1.00**	0.86	1.18	**1.04**	0.91	1.18
< 0.01	1	1	1	**1**	1	1	**1**	1	1
Respirable dust (mg/m^3^ p)									
> 1.0	**1.10**	0.96	1.26	0.98	0.77	1.26	**1.11**	0.89	1.39
0.1–1.0	**1.03**	0.94	1.12	0.94	0.82	1.07	**1.05**	0.91	1.22
< 0.1	**1**	1	1	1	1	1	**1**	1	1
Inhalable dust (mg/m^3^ s)									
> 5	**1.14**	0.94	1.39	0.90	0.63	1.29	***1.36***	***1.03***	***1.81***
0.1–5	**1.07**	0.91	1.26	0.92	0.69	1.23	***1.27***	***1.01***	***1.59***
< 0.1	**1**	1	1	1	1	1	***1***	1	1
Total dust (mg/m^3^ s)									
> 5	**1.08**	0.93	1.25	0.98	0.76	1.28	**1.13**	0.92	1.40
0.1–5	**1.02**	0.93	1.12	1.05	0.89	1.24	**1.06**	0.93	1.21
< 0.1	**1**	1	1	1	1	1	**1**	1	1
Respirable dust (mg/m^3^ s)									
> 1.0	1.05	0.91	1.22	0.84	0.64	1.09	**1.17**	0.94	1.45
0.1–1.0	0.98	0.86	1.11	0.80	0.64	1.00	**1.04**	0.87	1.24
< 0.1	1	1	1	1	1	1	**1**	1	1
Respirable quartz (mg/m^3^ s)									
> 0.05	1.04	0.90	1.20	1.04	0.80	1.35	**1.13**	0.92	1.40
0.01–0.05	1.09	0.99	1.20	1.08	0.91	1.29	**1.10**	0.96	1.27
< 0.01	1	1	1	1	1	1	**1**	1	1
PM 10 (mg/m^3^ s)									
> 2.5	**1.13**	0.92	1.40	0.85	0.59	1.24	***1.39***	***1.03***	***1.87***
0.1–2.5	**1.06**	0.87	1.28	0.77	0.55	1.09	**1.18**	0.90	1.54
< 0.1	**1**	1	1	1	1	1	**1**	1	1
PM 2.5 (mg/m^3^ s)									
> 2	**1.15**	0.97	1.37	0.90	0.66	1.23	**1.26**	0.98	1.62
0.1–2	**1.09**	0.96	1.24	0.92	0.73	1.15	**1.18**	0.98	1.41
< 0.1	**1**	1	1	1	1	1	**1**	1	1
PM 1 (mg/m^3^ s)									
> 0.5	**1.04**	0.89	1.22	0.87	0.66	1.15	1.08	0.86	1.35
0.1–0.5	**1.03**	0.94	1.13	1.07	0.90	1.26	1.11	0.97	1.28
< 0.1	**1**	1	1	1	1	1	1	1	1
Particle surface area (µm^2^/cm^3^ s)									
> 10	1.06	0.92	1.23	0.93	0.72	1.21	1.06	0.86	1.30
2–10	1.09	1.00	1.20	1.01	0.85	1.19	1.10	0.96	1.26
< 2	1	1	1	1	1	1	1	1	1
Particle number (nr/10^4^ cm^3^ s)									
> 1000	***1.11***	***1.00***	***1.24***	1.07	0.88	1.31	**1.11**	0.95	1.31
100–1000	***1.07***	0.97	1.18	0.96	0.80	1.15	**1.08**	0.93	1.25
< 100	***1***	1	1	1	1	1	**1**	1	1

*β* fixed effect by exposure class, 95% CI (confidence interval)

Bold values indicate exposure response, both significant and nonsignificant, and italic significant in the high exposure class

*p* personal sampling, *s* stationary sampling

*β*_2_ is the fixed effect of sampling time. *β*_3_ is the fixed effect of gender (male or female), with males serving as the reference category. *β*_4_ is the fixed effect of BMI, dichotomized by median, the highest level was used as the reference category. *β*_5_ is the fixed effect of smoking habits; categories considered were smoker, ex-smoker and lifetime non-smoker, with the latter being the reference category. *β*_6_ is the fixed effect of age dichotomized by median; the oldest group served as the reference category. *β*_7_ is the fixed effect of infections, with non-infection as the reference category. *β*_8_ is the fixed effect of blood group (0), with other blood groups as the reference category. *ε* is the residual.

The results of the mixed model analysis are presented as *β* values and 95% confidence intervals. The statistical significance threshold was *p* < 0.05. All analyses were performed with SPSS 22.0.

## Results

### Exposure

The 8-h TWAs for the respirable quartz exposures measured by personal sampling of the 85 participants ranged from 0.001 to 0.61 mg/m^3^, with a mean of 0.052 mg/m^3^. In contrast, the mean and maximum quartz exposures determined by stationary measurement were 0.026 mg/m^3^ and 0.26 mg/m^3^, respectively (Table 1). The highest personal exposures were observed in the core making, shake-out and fettling departments, for which the maxima were 0.33, 0.29 and 0.61 mg/m^3^, respectively (Table 2).

For the personal measurements of the 28 persons using respirators, we calculated adjusted exposure concentrations based on non-adjusted, average and background-adjusted respirable dust and quartz concentrations. The total average respirable quartz concentrations for these were 0.052, 0.031 and 0.022 mg/m^3^, respectively.

### Markers of inflammation and coagulation

The measured levels of biological markers of inflammation and coagulation in the subjects’ plasma and serum are presented in Table 3. These results were used in the development of the mixed model. The exposure–response analysis was based on personal exposure and stationary particle mass, number and surface area concentration measurements. Statistically significant increases in *β* values (representing the fixed effects of exposure) were observed, particularly in the highest exposure group, for SAA, fibrinogen and FVIII. The particulate fractions associated with these increases were inhalable dust, PM_2.5_, and PM_1_ for SAA; inhalable dust and PM_10_ for FVIII; and particle number concentration for fibrinogen. Non-significant exposure–responses were observed for IL-6, IL-8, SAA, CRP, vWF and FVIII with respect to respirable quartz (Tables 4, 5).

The mixed model analysis revealed that the levels of the inflammatory and coagulatory markers TNF, IL-1β, IL-6, IL-8 and FVIII in the second samples were significantly higher than those in the first samples, whereas the levels of SAA and vWF were significantly lower in the second samples. In addition, the levels of vWF and FVIII were significantly higher among subjects with blood group O than those with other blood groups. Workers reporting infections had significantly higher levels of IL-6, SAA, CRP and vWF than non-infected workers, with odds ratios of 1.6, 3.6, 3.5 and 1.5, respectively. Marker levels among smokers did not differ significantly from those among non-smokers.

## Discussion

The main finding of our study is that there are significant relationships between particle mass exposure metrics and the levels of SAA, fibrinogen and FVIII. There were also non-significant positive exposure–response relationships between several exposure metrics and other markers of inflammation and coagulation. In particular, respirable quartz exhibited (statistically non-significant) exposure–response patterns for IL-6, IL-8, SAA, CRP, vWF and FVIII. Most of the exposure–response patterns were related to mass-based exposure metrics, but positive exposure–responses were also observed for the particle surface area and particle number concentrations. These exposure–response patterns are similar to those reported in earlier studies.

To our knowledge, these are the first published data on particle exposure (particularly, quartz exposure) and inflammatory markers among foundry workers. A key strength of this study is the use of parallel blood sampling and the determination of multiple measures of exposure, including measures based on particle mass, surface area and number. The blood sampling protocol was designed to minimize the influence of diurnal variation: sampling was only conducted at the end of a shift to enable meaningful analysis of time trends and other determinants (Rudnicka et al. 2007). The participants’ blood groups were recorded because it has been suggested that exposure to occupational air pollutants may be associated with ischaemic heart disease among individuals with blood type O (Suadicani et al. 2002).

The study lacks a completely non-exposed referent group, because the use of such a group could introduce socioeconomic bias. In previous studies, we began air and blood sampling on the first day after the participants returned to work following a long vacation (Ohlson et al. 2010); this approach was not adopted here because of logistical difficulties. Our experience suggests that a single week’s absence from exposed work is insufficient to establish a true baseline level of inflammatory markers (Westberg et al. 2016).

However, we believe that even without establishing such a baseline, analyses of exposure–response relationships for inflammatory markers can reveal the important effects of particle exposure at levels close to occupational exposure limits.

Particle sampling and analysis and blood sampling were performed using standard methods at accredited laboratories. The overall quartz exposures and those for specific job titles at the three foundries are comparable to previously reported exposure measurements (Andersson et al. 2009). Some 30% of the participating workers used respirators during some of their working time; the duration of these periods was recorded for each worker. Three different measures of particulate exposure were computed for these workers: the non-adjusted measure (ignoring the effect of the respirator), an adjusted measure assuming zero exposure while the respirator was worn and uncorrected exposure for the remainder of the day, and a background-adjusted measure assuming zero exposure while the respirator was worn and exposure at the background level for the worker’s department otherwise. We consider the latter measure to be the most reliable. For reasons of sensitivity, mixed model analyses were performed using all three adjusted and non-adjusted measures of exposure. There were no appreciable differences between the resulting exposure–response analyses.

Workers in foundry environments may also be exposed to chemical compounds that may be linked to cardiovascular disease—notably, polycyclic aromatic hydrocarbons (PAHs) originating from incomplete combustion of organic substances, which may be emitted in the melting, casting and shake-out areas. A review of studies on PAHs in iron foundries found that the reported air concentrations of B(a)P ranged from 0.01 to 2.1 µg/m^3^ for melting, casting and shake-out operations (Lindstedt and Sollenberg 1982).

We used log-linear modelling to study the exposure–response relationships between particle exposure and inflammatory markers. Determinants assumed to influence or confound the exposure–response relationships were included in the models, and we also determined exposure responses for subjects with and without infections, using the latter as positive controls. Workers reporting infections exhibited significantly elevated concentrations of IL-6, SAA, CRP and vWF. The observation of elevated levels of markers related to ongoing infections among this group indicates that our model design was valid for studying inflammatory responses.

CRP is one of the most sensitive acute-phase reactants and is widely used as a clinical biomarker. A review by Li et al. (2012) highlighted three occupational studies in which this marker was used. Among a group of 37 welders, significantly elevated levels of CRP were observed for both smokers and non-smokers exposed to median PM_2.5_ concentrations of 1.69 mg/m^3^ (25th to 75th percentile range 0.89–2.44 mg/m^3^) (Kim et al. 2005). A similar significant increase was observed among 37 steel production workers exposed to fine particles (PM_1_; median exposure 0.0036 mg/m^3^, range 0.0017–0.033 mg/m^3^) and coarse particles (PM_10_; median exposure 0.162 mg/m^3^, range 0.071–1.2 mg/m^3^) (Bonzini et al. 2010). An analysis of 73 subjects employed in welding, cutting, grinding and in iron and aluminium foundries conducted by our group identified a statistically significant increase in CRP levels associated with a mean total dust exposure of 0.93 mg/m^3^ (range 0.034–6.4 mg/m^3^) (Ohlson et al. 2010). Finally, a study conducted by our group on 67 workers in the pulp and paper industry showed that several mass-based exposure measures (including inhalable particulate matter, total dust and PM_10_) were associated with statistically significant increases in CRP levels, especially among the high exposure subject group. The subjects’ mean inhalable dust exposure was 0.30 mg/m^3^. However, there was no detectable exposure response for the respirable fractions or the particle surface area air concentration (Westberg et al. 2016). Multiple occupational studies have thus identified increased CRP levels among workers exposed to dust concentrations of the same order of magnitude as those reported here (i.e., mean or medians between 0.1 and 1.7 mg/m^3^).

Fibrinogen levels exhibited exposure–response patterns with respect to all of the mass-based exposure measures other than the surface area and particle number concentrations among the foundry workers. Elevated fibrinogen levels were observed in both the high (> 0.05 mg/m^3^) and medium (0.03–0.05 mg/m^3^) quartz exposure groups, and the high (> 1.0) and medium (0.1–1.0 mg/m^3^) respirable dust exposure groups. In our first study on particulate exposure in different industrial settings (Ohlson et al. 2010), we observed no exposure response for particulate matter and fibrinogen on day 1 of the investigation, but an increase was noted on day 2. This may be because the sampling campaign in that study began immediately after the workers had returned from their summer vacation, and the fibrinogen response was comparatively slow (Saber et al. 2014). However, our study on the pulp and paper industry revealed significant exposure–response relationships between fibrinogen and several exposure measures including the inhalable dust (for which the mean concentration was 0.3 mg/m^3^, with a range of 0.005–3.3 mg/m^3^), PM_10_, respirable dust, particle surface area and particle number concentrations (Westberg et al. 2016). Other studies have revealed significant increases in fibrinogen levels associated with high concentrations of dust containing endotoxins (> 15 mg/m^3^) (Sjögren et al. 1999). However, significant reductions in fibrinogen levels have been reported among welders (Kim et al. 2005).

SAA, an acute-phase protein comparable to CRP, has not been studied in occupational environments, but there have been some studies on its responses in ambient air (Ruckerl et al. 2006). In our study on the pulp and paper industry, we observed significant increases in SAA levels (similar to those observed for CRP) associated with most mass-based exposure measures, particularly among the high exposure subject group. However, such associations were not observed for the particle surface area or particle number concentrations. SAA may play a role in atherosclerosis because it has been casually related to plaque formation in the aorta in animal studies (Vogel and Cassee 2018).

Factor VIII is a marker of inflammation and haemostasis and also a risk factor for coronary heart disease (Folsom et al. 1997; Haverkate 2002). Relations between ambient air pollutant (PM_10_) (Liao et al. 2005) and wood smoke exposures (Barregård et al. 2006) and plasma levels of factor VIII have been observed previously. We found significant relations between both inhalable dust and PM_10_ and increases of factor VIII, indicating an increased risk of coagulation and consequently disease by this exposure.

In our first study on particle exposure during welding, cutting, grinding and other foundry operations, we identified exposure–response relationships between particulate matter and IL-6 in workers returning from long vacations who were exposed to average total dust levels of 0.93 mg/m^3^ (Ohlson et al. 2010). However, the exposure–response relationships observed for IL-6 and other cytokines (IL-1β, IL-10 and IL-8) in our study on the pulp and paper industry were weaker and rarer (Westberg et al. 2016). The comparative weakness of these responses can be attributed to differences in study design, particularly the fact that the exposure-free period prior to sampling was much longer in the first study than in the second (in which the exposure-free period was only 1 week long). In this work, exposure–response relationships were tentatively identified for IL-6 and IL-8 with respect to several mass-based exposure measures as well as the particle surface area and particle number concentrations in air.

Elevated levels of IL-6 and IL-8 were found among nanoparticle-exposed woodworkers and metalworkers when compared to a control group of office workers (Kurjane et al. 2017). Swine farmers and volunteers exposed to about 20 mg/m^3^ of organic dust for part of a work shift also showed increased levels of IL-6 and IL-8, and the increase was more pronounced among the non-farmers (Palmberg et al.^2002^). Similar findings were obtained when volunteers were exposed to zinc oxide at 5 mg/m^3^: the IL-6 levels induced by short-term exposure were three times higher than those observed in subjects who had experienced long-term exposure. This suggests that an adaptation or low-grade inflammatory process occurs after prolonged exposure, with comparatively low levels of cytokines (Fine et al. 2000).

In this work, we defined three levels of quartz exposure based on our objective of studying inflammatory responses to quartz concentrations on the same order of magnitude as current occupational exposure limits. The highest exposure group considered in this work comprised individuals with exposures above 0.05 mg/m^3^, which is the exposure limit recommended by the EU (SCOEL 2002), and twice the ACGIH-recommended TWA-TLV of 0.025 mg/m^3^ (ACGIH 2018). However, these OELs are primarily intended to prevent silicosis; the limits required to minimize the risk of cardiovascular disease may be different.

Two recently published papers quantitatively examined the relationship between quartz dust exposure and cardiovascular mortality and disease. Among Chinese metal mine and pottery workers, the risk of ischaemic heart disease was almost doubled by a cumulative quartz exposure of > 0.67 mg/m^3^ year for workers exposed to ≤ 0.05 mg/m^3^ of respirable quartz. However, among workers exposed to respiratory quartz levels of ≤ 0.10 mg/m^3^, a significant risk of pulmonary heart disease occurred when the cumulative exposure exceeded > 0.86 mg/m^3^ year (Liu et al. 2017). A significant increase in cardiovascular mortality was observed in a cohort of Swedish foundry workers, which was linked to respiratory quartz on the basis of an exposure–response analysis. The highest cumulative exposure to respirable quartz in this cohort was > 0.65 mg/m^3^ year (Fan et al. 2018). These cumulative exposures resulting in excess risks would correspond to a daily average exposure of around 0.02 mg/m^3^ over a period of 40 years. We observed significant exposure–response relationships between inflammatory markers and quartz levels in the highest exposure group of our cohort, whose exposure was > 0.05 mg/m^3^. Exposure at this level may thus be associated with an increased risk of cardiovascular disease.

Time series analyses of urban air pollutants indicate that cardiovascular mortality increases by 0.4% for every 10 µg/m^3^ increase in PM_10_ levels (Brook et al. 2010). The geometric mean (GM) PM_10_ air concentration (1.1 mg/m^3^) determined based on our stationary measurements was used to estimate the potential increase in cardiovascular risk among our foundry workers. Assuming a background PM_10_ concentration of 50 µg/m^3^ exposure at the measured geometric mean would theoretically increase cardiovascular mortality by 42%, calculated as (1100 − 50)/10 × 0.4%. This increase is of the same order as magnitude, as reported for the Swedish SMR 1.41 cohort (Fan et al. 2018).

Other chemical agents suspected to cause cardiovascular disease that are known to occur in the foundry environment include PAHs, and benzo(a)pyrene in particular (SBU 2017). Occupational exposures to these compounds are generally low when tar-free binders are used for moulding; high PAH levels typically only occur in the vicinity of melting, casting and shake-out operations. This pattern was not observed for quartz exposure levels in our study, so it is unlikely that the exposure–response patterns observed in this work can be attributed to benzo(a)pyrene exposure.

## Conclusion

We have measured air exposure levels in terms of particle mass, number and surface area concentrations as well as the levels of selected inflammatory markers in workers at Swedish iron foundries, focusing on the effects of quartz dust exposure. When expressed as 8-h time-weighted averages (TWA), the average participating worker was exposed to a respirable dust concentration of 0.85 mg/m^3^ (with a range of 0.062–9.7 mg/m^3^); the corresponding value for respirable quartz was 0.052 mg/m^3^, with a range of 0.001–0.61 mg/m^3^. Statistically significant increased levels of inflammation and coagulation markers, namely SAA, fibrinogen and FVIII, were observed in the highest exposure groups for some exposure measures. In addition, statistically non-significant exposure–response patterns were observed for respirable quartz and the markers IL-6, IL-8, SAA, CRP, vWF and FVIII. These relationships between particle exposure and inflammatory markers may indicate an increased risk of cardiovascular disease.
